# Impaired Nitric Oxide Mediated Vasodilation In The Peripheral Circulation In The R6/2 Mouse Model Of Huntington’s Disease

**DOI:** 10.1038/srep25979

**Published:** 2016-05-16

**Authors:** Andrew D. Kane, Youguo Niu, Emilio A. Herrera, A. Jennifer Morton, Dino A. Giussani

**Affiliations:** 1 Department of Physiology, Development & Neuroscience, University of Cambridge, Downing Street, Cambridge, CB2 3EG, UK

## Abstract

Recent evidence shows that the Huntington’s disease (HD) extends beyond the nervous system to other sites, including the cardiovascular system. Further, the cardiovascular pathology pre-dates neurological decline, however the mechanisms involved remain unclear. We investigated in the R6/2 mouse model of HD nitric oxide (NO) dependent and independent endothelial mechanisms. Femoral artery reactivity was determined by wire myography in wild type (WT) and R6/2 mice at 12 and 16 weeks of adulthood. WT mice showed increased endothelial relaxation between 12 and 16 weeks (R_max_: 72 ± 7% *vs*. 97 ± 13%, P < 0.05). In contrast, R6/2 mice showed enhanced endothelial relaxation already by 12 weeks (R_max_ at 12w: 72 ± 7% *vs*. 94 ± 5%, WT *vs*. R6/2, P < 0.05) that declined by 16 weeks compared with WT mice (R_max_ at 16w: 97 ± 13% *vs*. 68 ± 7%, WT *vs*. R6/2, P < 0.05). In WT mice, the increase in femoral relaxation between 12 and 16 weeks was due to enhanced NO dependent mechanisms. By 16 weeks of adult age, the R6/2 mouse developed overt endothelial dysfunction due to an inability to increase NO dependent vasodilation. The data add to the growing literature of non-neural manifestations of HD and implicate NO depletion as a key mechanism underlying the HD pathophysiology in the peripheral vasculature.

Huntington’s disease (HD) is a progressive, devastating and currently incurable neurodegenerative genetic disorder caused by a mutation in the *HTT* gene that codes for huntingtin^1^. The overt neurological signs and symptoms have previously directed investigators towards the nervous system as the primary site of pathology. Huntingtin, nevertheless, is expressed in many non-neuronal tissues although its extra-neuronal effects, including those in the cardiovascular system, are poorly characterised^2^,^3^,^4^,^5^. Whilst current evidence does not clearly support an increase in cardiovascular related mortality in patients with HD^6^, it is a common cause of death in patients with HD^7^ and many clinical studies have shown a significant shift in their autonomic cardiovascular control towards increased sympathetic dominance with reduced parasympathetic influences^8^,^9^,^10^. Interestingly, we and others have shown, in the R6/2 mouse model of HD, that significant cardiac dysfunction is already present at earlier stages of neurological decline^11^,^12^,^13^. The R6/2 mouse has a smaller cardiac stroke volume, ejection fraction and cardiac output *in vivo* from 12 weeks of age, a stage where neurological signs are largely absent but the animals are failing to gain weight compared to WT mice^13^,^14^. When examined *ex vivo*, the R6/2 mouse is unable to generate the left ventricular pressure of its age matched WT counterpart at 16 weeks of age, an age by which the animals are clearly ‘symptomatic’^11^. Further, *in vivo* the R6/2 heart is unable to respond positively to β_1_ adrenergic stimulation^14^.

Such overt derangements in cardiac function in the R6/2 mouse may be expected to be associated with peripheral vascular dysfunction. To date, however, only one study using the R6/1 mouse model of HD has examined this possibility^15^. In that study, Rahman *et al*. reported that there was no effect on dilator capacity in the aorta, although there was some evidence of vascular constrictor dysfunction in mesenteric and caudal vessels, which developed following the onset of neurological symptoms. No study has determined changes in vasodilator capacity in peripheral resistance vessels in HD, or whether any impairment is mediated via endothelium-dependent or independent mechanisms. Here, we tested the hypothesis that the R6/2 mouse shows nitric oxide (NO)-dependent endothelial dysfunction in peripheral resistance circulations, which pre-dates the neurological manifestations of HD. Given that HD patients and R6/2 mice display evidence of sympathetic autonomic dominance^8^,^9^,^10^, we further investigated femoral artery α_1_-adrenergic constrictor function in addition to NO-dependent and independent dilator function in the R6/2 mouse at 12 and 16 weeks of age, corresponding to early symptomatic and established impaired neurological stages of the disease process, respectively^16^.

## Methods

### Animals

All procedures were performed in accordance with the UK Animals (Scientific Procedures) Act 1986 and were overseen by the United Kingdom Home Office. Further local ethics committee approval was obtained from the University of Cambridge. Only male mice were studies to control for sex differences. Male mice were taken from a colony of R6/2 transgenic mice established in the Department of Pharmacology, University of Cambridge, and maintained by backcrossing onto CBA × C57BL6N F1 female mice^17^. Genotyping and CAG repeat length measurement were carried out by Laragen (Los Angeles, CA, USA) as described previously^18^. The transgenic mice used in this study had a mean CAG repeat length of 242 ± 1 (range 237–251). Mice were kept in home cages comprising single sex, single genotype groups of ten. All of the mice lived in an enhanced environment with increased amounts of bedding and nesting materials. Clean cages were provided twice weekly with grade 8/10-corncob bedding, and finely shredded paper for nesting. The mice were maintained on a 12:12 hour light/dark (LD) cycle, at a temperature of 21–23 °C and a humidity of 55 ± 10%. The mice had *ad libitum* access to water and dry laboratory food (RM3(E) rodent pellets, Special Diet Services, Witham, UK). In addition, once a day, a mash was prepared by soaking 100 g dry food in 230 ml water until the pellets were soft and fully expanded. The mash was placed on the cage floor, improving access to food and water for the R6/2 transgenic mice. This feeding regime has been shown previously to be beneficial^19^.

### *In vitro* wire myography

Segments of femoral arteries were mounted on a wire myograph, as previously described in detail^20^,^21^. In brief, under a bifocal dissecting microscope (Brunel Microscopes Ltd., Wiltshire, UK), the first branch from the femoral artery of the left hind limb was excised and place in ice cold saline solution. The vessel was carefully cleaned of excess connective tissue and cut to a 2 mm long ring. Two 40 μm diameter stainless steel wires were carefully threaded through the lumen of the femoral sections, maintaining the endothelium intact. The wires were then placed between the mounting support jaws of a 4-chamber small-vessel wire myograph (Multi Wire Myograph System 610M; DMT, Aarhus, Denmark) containing warmed oxygenated Krebs buffer (NaCl 118.5 mM, Fisher Scientific; KCl 4.75 mM, Sigma; MgSO_4_.7H_2_0 1.2 mM, Sigma; KH_2_PO_4_ 1.2 mM, Sigma; NaHCO_3_ 25.0 mM, Sigma; CaCl_2_ 2.5 mM, Sigma; glucose 11.1 mM, Sigma, UK; bubbled continuously with 95% O_2_/5% CO_2_ mix, 37 °C).

Force data from the myograph were recorded at 4 Hz (Labchart 6.0, Powerlab 8/30; AD Instruments, Chalgrove, UK), and each vessel was standardized to an optimal working tension of 100 mmHg^22^. Both vasoconstrictor and vasodilator function was assessed in the vessels. All vessels were contracted with increasing concentrations of potassium chloride (KCl, 4.75–100 mM). Vasoconstrictor function to phenylephrine (PE: 10^−9^ to 10^−5^ M; Sigma Aldrich, Poole, UK) and vasodilator responses to sodium nitroprusside (SNP: 10^−9^ to 10^−4^ M; Sigma Aldrich) and endothelium-dependent vasodilatation with the acetylcholine analogue methacholine (10^−9^ to 10^−4.5^ M; Sigma Aldrich) were assessed. Vasoconstrictor responses to phenylephrine were normalised to the response of KCl at 40 mM for each vessel to standardise for any differences in muscle mass. Vasodilator responses with methacholine were assessed with no additive in the Krebs and separately with N^(G)^-nitro-L-arginine methyl ester (L-NAME, an endothelial nitric oxide synthase inhibitor; Sigma Aldrich, Poole, UK). All vasodilator responses were assessed with the vessels pre-contracted with PE (10^−5^ M) at a stable plateau. Responses were recorded for 2 minutes after each dose, whereupon the next cumulative dose was given. Vessels were repeatedly washed with Kreb’s solution and allowed to equilibrate for at least 20 minutes between different concentration-response curves.

### Analysis and statistics

Individual vessel responses were fitted to a Boltzmann sigmoidal (K^+^) or non-linear log (agonist) *vs*. response (PE, SNP, MetCh; GrpahPad Prism), as previously described In detail^23^. Maximal responses for each vessel and drug were taken from the curve fit value. Sensitivity was defined as the concentration of agonist required to elicit fifty percent of the maximal response expressed as either the EC_50_ (Excitatory concentration to achieve 50% maximal response) or pD_2_ (where pD_2_ = −log_10_ EC_50_)^24^. An index of total endothelial relaxation (NO dependent + NO independent) was calculated in each vessel by calculating the area above the methacholine relaxation curve between 10^−9^ and 10^−4^ M as previously described^21^,^23^. An index of NO independent relaxation was calculated by the area above the methacholine relaxation curve in the presence of L-NAME and NO dependent relaxation was calculated as the difference between total and NO independent relaxation^21^,^23^. All data were compared statistically by Two-Way ANOVA followed by *post hoc* Bonferroni test where significant effects of WT vs R6/2, 12 vs 16 weeks of age or an interaction were found. Significance was accepted when P < 0.05 (SigmaStat 2.0; SPSS Inc., Chicago, USA).

## Results

### Femoral artery vasodilator function

**Figure 1:**
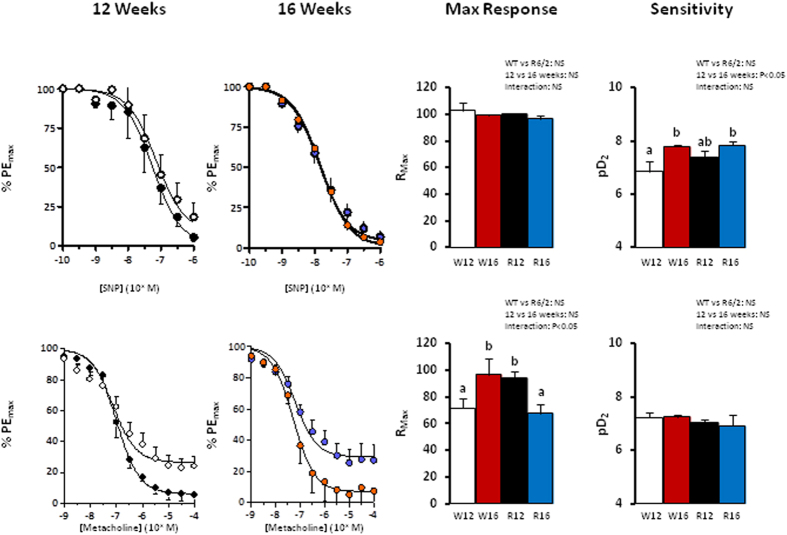
Femoral artery vasodilator function in the R6/2 mouse. Values are mean ± S.E.M. for the concentration-response curve maximal response and sensitivity to sodium nitroprusside (SNP) and methacholine in femoral arteries of 12 week old WT (○, W12, n = 9), 12 week old R6/2 (●, R12, n = 9), 16 week old WT (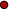, W16, n = 9) and 16 week old R6/2 (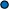, R16, n = 7) mice. Concentration-response curves were analysed using an agonist-response best-fit line. The maximal relaxant response (%PE_max_) was expressed as percentage of the contraction induced by phenylephrine and the vascular sensitivity was expressed as pD_2_ (−logEC_50_). Different letters, a and b, represent significantly different responses between groups, P < 0.05. Two-way ANOVA with *post-hoc* Bonferroni Test.

**Figure 2:**
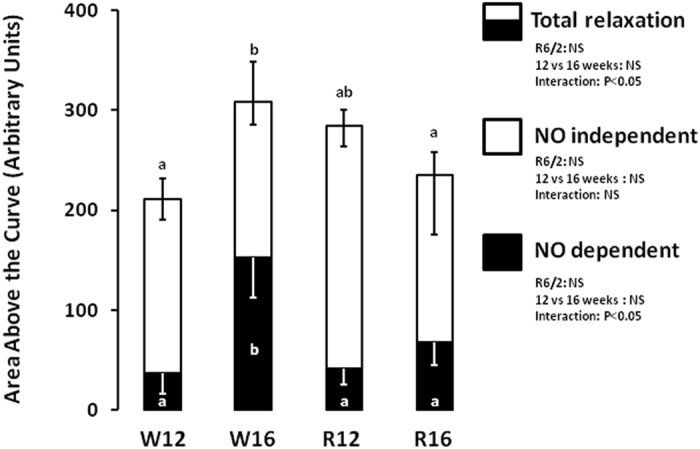
Relative contributions to endothelial dependent relaxation in the R6/2 mouse. The data show the area above the relaxation curve in response to methacholine. The total bar represents total vasodilator capacity. NO independent relaxation is the area above the curve in the presence of the NOS inhibitor L-NAME and NO dependent is the difference between total vasodilator capacity and NO independent relaxation. Experiments performed in femoral arteries of 12 week old WT (W12, n = 8), 12 week old R6/2 (R12, n = 6), 16 week old WT (W16, n = 8) and 16 week old R6/2 (R16, n = 5) mice. Different letters, a and b, represent significantly different responses between groups, P < 0.05. Two-way ANOVA with *post-hoc* Bonferroni Test.

Both SNP and methacholine led to dose dependent vasodilation in femoral arteries from WT and R6/2 mice at 12 and 16 weeks of age in vessels pre-contracted with phenylephrine (Fig. 1, P < 0.05). There were no significant differences in the maximal response to SNP in any group, suggesting no differences in vascular smooth muscle potential to vasodilate (Fig. 1). WT mice showed an increase in endothelial dependent relaxation in response to methacholine between 12 and 16 weeks of adulthood (Figs 1 and 2). In contrast, R6/2 mice showed an increase in endothelial dependent relaxation already at 12 weeks and a significant decline by 16 weeks compared to WT mice at corresponding ages (Fig. 1). When NO dependent and independent mechanisms underlying the endothelium-mediated relaxation were investigated, the increase in vasodilator capacity in the WT mice at 16 weeks was due to an increase in NO-dependent relaxation (Fig. 2). In contrast, this increase in NO-dependent endothelial mediated relaxation in the femoral resistance vessel with increasing age was not present in the R6/2 mouse (Fig. 2).

### Femoral artery vasoconstrictor function

**Figure 3:**
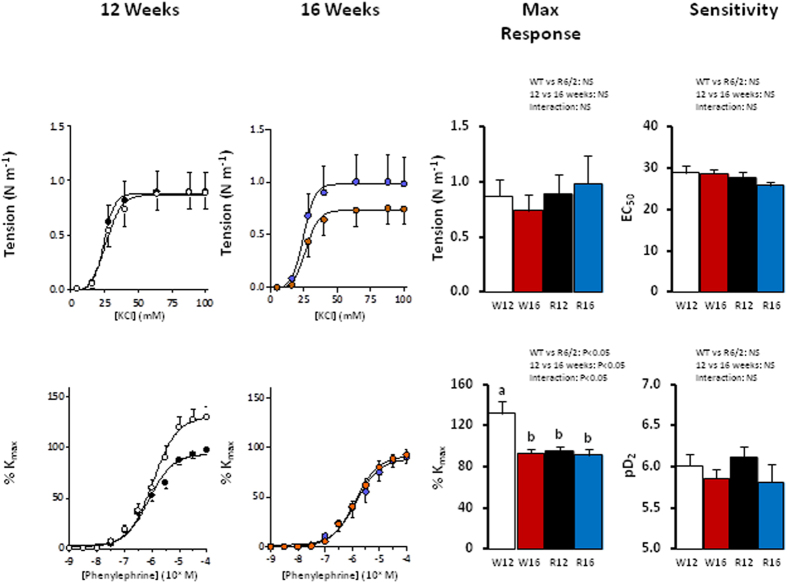
Femoral artery vasoconstrictor function in the R6/2 mouse. Values are mean ± S.E.M. for the concentration-response curve maximal response and sensitivity to potassium chloride (KCl) and phenylephrine in femoral arteries of 12 week old WT (○, W12, n = 9), 12 week old R6/2 (●, R12, n = 9), 16 week old WT (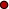, W16, n = 9) and 16 week old R6/2 (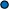, R16, n = 7) mice. Concentration-response curves were analysed using an agonist-response best-fit line. The maximal contractile response and sensitivity to each drug are shown. Different letters, a and b, represent significantly different responses between groups, P < 0.05. Two-way ANOVA with *post-hoc* Bonferroni Test.

At both 12 and 16 weeks of age, application of KCl and PE led to dose dependent increases in femoral arterial tension in both WT and R6/2 mice (Fig. 3). WT mice showed a decrease in the maximal vasoconstrictor response to PE with advancing age from 12 to 16 weeks. In contrast, the R6/2 mouse showed impaired maximal constrictor responses to PE already at 12 weeks compared to WT, which was not worsened further by 16 weeks (Fig. 3). There were no differences in the maximal response or sensitivity to KCl.

## Discussion

The striking neurological manifestations of HD have led investigators to focus on the nervous system in search of the underlying pathology and management of this disorder^1^. However huntingtin, the mutated protein, is expressed extensively at many extra-neuronal sites including the heart and circulatory system^3^. Here, we show that endothelial function is significantly altered in peripheral resistance arteries of the R6/2 mouse from 12 weeks of age and grossly impaired by 16 weeks. In contrast to WT mice, which showed an increase from 12 to 16 weeks of age in endothelial-mediated relaxation via increased NO-dependent mechanisms, the R6/2 mouse failed to recruit NO-dependent pathways and increase relaxation from 12 to 16 weeks of age. Furthermore, at 12 weeks of age, before the onset of overt neurological signs in this model, the R6/2 mice displayed significant enhancement of endothelial-dependent dilation and impaired α_1_ adrenergic vasoconstrictor responses. Collectively, the data support the hypothesis tested and show for the first time significant peripheral vascular endothelial dysfunction due to impaired NO-dependent mechanisms in a mouse model of HD. We also provide evidence for the peripheral vascular dilator and constrictor dysfunction preceding the onset of overt neurological symptoms.

Several mechanisms may be involved in triggering dysfunction in peripheral resistance circulations. In the present study, our data suggest that in the R6/2 mouse model of HD alterations in endothelial function and in NO biology may contribute to the pathophysiological decline. It is now understood that the cellular oxidant *milieu* is an important modulator of peripheral vascular resistance^25^. In resistance vessels, the balance between superoxide (^.^O_2_^−^) and NO has important effects on endothelial function, both under physiological regulation and in disease pathophysiology. Under physiological conditions, the ratio of vascular NO:^.^O_2_^−^ is an important determinant of vascular tone. Therefore, during conditions in which NO production outweighs ^·^O_2_^−^ production, the increase in the NO:^.^O_2_^−^ ratio promotes vasodilation^25^,^26^. When vascular ^·^O_2_^−^ production outweighs NO, this leads to vascular constriction and an increase in peripheral vascular resistance^25^,^26^. Under pathological conditions, excess generation of reactive oxygen species and oxidative stress may therefore decrease NO bioavailability and promote endothelial dysfunction. Interestingly, it is already known that the huntingtin protein promotes oxidative stress^27^. Indeed, reactive oxygen species mediated mitochondrial DNA damage^28^, general protein nitrosylation^27^ and lipid peroxidation^27^,^29^ are all found in HD. Further, mitochondrial dysfunction has been strongly associated with a rise in oxidative stress in HD^27^,^30^. Specifically the R6/2 mouse displays dysfunction of voltage-dependent anion channel 1^31^, which is a key component of the mitochondrial permeability transition pore and a regulator of superoxide anion leakage from mitochondria^30^,^32^. Collectively, past and present data therefore suggest impaired NO signalling as one mechanism underlying the inability of the R6/2 mouse to increase femoral artery vasodilation from 12 to 16 weeks of adult age. The enhanced femoral artery dilation at 12 weeks of age in the R6/2 mouse may represent a compensatory response to impaired NO bioavailability, an adaptive response which is later decompensated as NO-dependent vasodilation fails at 16 weeks.

Additional data in the present study show no alteration in peripheral vasoconstrictor function in response to potassium between the groups but a markedly impaired femoral artery α_1_-mediated constrictor response already at 12 weeks of life in the R6/2 mouse, akin to that measured in the WT mouse much later at 16 weeks of age. It is known that HD patients show a sympathetically dominated autonomic nervous system phenotype^8^,^9^,^10^. As well as driving sympathetically dominant patterns of heart rate variability and structural and functional abnormalities in the heart, it is possible that the autonomic sympathetic outflow to the vasculature is also enhanced in the R6/2 mouse. In the R6/2 mouse, it has been suggested that the cardiomyopathy is caused by altered central autonomic pathways, since neither mutant huntingtin aggregates nor a HD-specific transcriptional dysregulation was identified in cardiac tissue, even at the end stage of disease^13^. While aggregates of mutant huntingtin are a hallmark of HD brain, their presence (or lack thereof) does not always correlate with pathological cellular pathology. Therefore, it is possible that cell autonomous pathology in cardiac tissue is present. This remains to be investigated directly. Nevertheless, alterations in the metabolic and endocrine *milieu* which impact on vascular function are established in HD. For example, plasma cortisol levels are raised in both the R6/1 and R6/2 mouse model of HD, an effect which would also contribute to enhance α_1_-adrenergic receptor mediated peripheral vasoconstriction^15^,^33^,^34^. In the R6/1 model of HD, plasma noradrenaline levels are reported to be 4–5x higher than WT animals at 7 months of age, when the R6/1 animal shows advanced HD-like phenotype^35^. Sustained increases in sympathetic nervous system activity and in circulating catecholamine and glucocorticoids are strongly associated with endothelial dysfunction and cardiovascular disease in general^36^. Therefore, the accelerated loss of the α_1_-adrenergic receptor mediated reactivity in peripheral resistance vessels of the R6/1 mouse may represent an adaptive response, down-regulating α_1_-adrenoreceptor responsiveness to an increased sympathetic drive, which is exacerbated by increased circulating plasma glucocorticoid concentrations. Interestingly, in human patients with Huntington’s disease there appear to be no substantial differences in hypothalamic-pituitary-adrenal axis control^37^.

Finally, changes in sympathetic stimulation combined with expression of the huntingtin protein may lead to structural alterations in resistance vessels. Defects have been reported in neurovascular tissue from humans with HD and in mouse models at the level of the blood brain barrier^38^, although in the R6/1 mouse there is no apparent peripheral vascular remodelling in mesenteric or caudal arteries^15^. In support of this, in the present study, responses to potassium chloride, which reflect a global measure of vascular smooth muscle structure, mass and function, were not altered in the R6/2 compared to WT mouse.

### Translational perspective

Clinical studies show clearly that cardiovascular dysfunction is present in HD. Furthermore, the recent body of evidence in animal models of HD confirms that the systemic over-expression of huntingtin leads to premature cardiac and vascular dysfunction. Despite this, in clinical practice, HD is still perceived almost exclusively as a neurological disorder due to the overt neurological manifestations, with little or no clinical appreciation for extra-neuronal sites of expression of the disease. Using an established mouse model of HD, here we show that endothelial NO-dependent function is markedly impaired in the peripheral vasculature. Collectively, past and present data therefore support increasing attention to the monitoring of the cardiovascular system in patients with HD. In particular, investigation of whether treatment with drugs that enhance NO bioavailability already in common clinical use, such as statins, may protect peripheral vascular function in HD patients is warranted.

## Additional Information

**How to cite this article**: Kane, A. D. *et al*. Impaired Nitric Oxide Mediated Vasodilation In The Peripheral Circulation In The R6/2 Mouse Model Of Huntington’s Disease. *Sci. Rep*. **6**, 25979; doi: 10.1038/srep25979 (2016).
